# Population genomics of emerging *Elizabethkingia anophelis* pathogens reveals potential outbreak and rapid global dissemination

**DOI:** 10.1080/22221751.2022.2132880

**Published:** 2022-11-04

**Authors:** Shaohua Hu, Hao Xu, Xiaohua Meng, Xiangxiang Bai, Junli Xu, Jinru Ji, Chaoqun Ying, Yunbo Chen, Ping Shen, Yunxiao Zhou, Beiwen Zheng, Yonghong Xiao

**Affiliations:** a State Key Laboratory for Diagnosis and Treatment of Infectious Diseases, Collaborative Innovation Center for Diagnosis and Treatment of Infectious Diseases, The First Affiliated Hospital, College of Medicine, Zhejiang University, Hangzhou, People’s Republic of China; b Bioinformatics Institute, Novogene Bioinformatics Technology Co., Ltd, Beijing, People’s Republic of China; c Department of Obstetrics & Gynecology, The First Affiliated Hospital, College of Medicine, Zhejiang University, Hangzhou, People’s Republic of China; d Department of Structure and Morphology, Jinan Microecological Biomedicine Shandong Laboratory, Jinan, People’s Republic of China

**Keywords:** *Elizabethkingia anophelis*, genome sequencing, phylogenetic structure, outbreak infection, global transmission

## Abstract

*Elizabethkingia anophelis* is an emerging species and has increasingly been reported to cause life-threatening infections and even outbreaks in humans. Nevertheless, there is little data regarding the *E. anophelis* geographical distribution, phylogenetic structure, and transmission across the globe, especially in Asia. We utilize whole-genome sequencing (WGS) data to define a global population framework, phylogenetic structure, geographical distribution, and transmission evaluation of *E. anophelis* pathogens. The geographical distribution diagram revealed the emerging pathogenic bacteria already distributed in various countries worldwide, especially in the USA and China. Strikingly, phylogenetic analysis showed a part of our China original *E. anophelis* shared the same ancestor with the USA outbreak strain, which implies the possibility of localized outbreaks and global spread. These closer related strains also contained ICEEaI, which might insert into a disrupted DNA repair *mutY* gene and made the strain more liable to mutation and outbreak infection. BEAST analysis showed that the most recent common ancestor for ICEEaI *E. anophelis* was dated twelve years ago, and China might be the most likely recent source of this bacteria. Our study sheds light on the potential possibility of *E. anophelis* causing the large-scale outbreak and rapid global dissemination. Continued genomic surveillance of the dynamics of *E. anophelis* populations will generate further knowledge for optimizing future prevent global outbreak infections.

## Introduction

*Elizabethkingia* is a genus of Gram-negative, aerobic, oxidase-positive, weakly indole-positive, non-fermenting, and non-motile bacillus belonging to the family *Flavobacteriaceae*. It can be widely found in natural environments such as soil and water [1,2]. Presently, *Elizabethkingia* has been mainly divided into six species, namely *Elizabethkingia meningoseptica*, *Elizabethkingia anophelis*, *Elizabethkingia miricola*, *Elizabethkingia ursingii*, *Elizabethkingia bruuniana*, and *Elizabethkingia occulta* [3,4]. Notably, *E. anophelis* is an emerging species and has increasingly been reported to cause life-threatening infections in humans. The first case attributed to *E. anophelis* was documented in the Central Africa Republic in 2011[5]. It was reported that this microorganism could contribute to severe neonatal meningitis, bacteraemia, eye infection, and respiratory disease [5–11]. Moreover, it may be shocking that some outbreaks (in Singapore, Hong Kong, Taiwan, the USA, and South Korea) of *E. anophelis*-associated infections have been described recent years [8–10,12–15].

Interests, in previous studies, many *E. anophelis* isolates have been misidentified as *E. meningoseptica*, and the prevalence of *E. anophelis* may therefore be underestimated [4,8,9]. For instance, 51 strains of mass spectrum identification *Elizabethkingia* isolates [*E. Meningoseptica* (49), *E. Meningoseptica*/ *E. miricola* (1), *E.miricola* (1)], were reidentified as *E. anophelis* bacteria [16]. Moreover, recent research showed that 72 previously isolated clinical species of *Elizabethkingia* were reidentified as *E. anophelis* by 16S rRNA gene sequencing [17].

There are two prominent peculiarities of *E. anophelis* infections: multi-drug resistance and high fatality rate [4,7,9,17], both threaten people’s health. A representative example is an outbreak in Wisconsin, USA. From the end of 2015 to June 2016, 65 cases had been identified as *E. anophelis* infections, and the arresting case fatality rate was reported to top 31% (20/65) [9,18].

In contrast to other familiar bacteria, the epidemiology, transmission, and evolutionary mechanism of *E. anophelis* were less well understood. The unknown pathogenesis mechanisms and multi-drug resistance limited available genomic information and lack of effective therapeutic regimens challenging the management of *E. anophelis* infections [5,7–9,19,20]. Further work to elucidate the above issues of this emerging pathogen may help improve the clinical management of illness. There is a paucity of *E. anophelis* data regarding geographical distribution, phylogenetic structure, and transmission globally, especially in Asia. Previously, we had collected 197 *E. anophelis* strains from different provinces in China. Then, we completed the genome sequencing and downloaded almost all the genomes of this bacteria in the National Center of Biotechnology Information (NCBI) databases. We utilize these WGS data to define a global population framework, phylogenetic structure, geographical distribution and transmission evaluation. Our analysis indicates there may be a potential outbreak and rapid global dissemination of *E. anophelis*.

## Materials and methods

### Study design and bacterial isolates

A longitudinal survey of *E. anophelis* isolated from clinical samples from the Strain Library of the First Affiliated Hospital of Zhejiang University School of Medicine between January 2010 and April 2019 was performed. Some bloodstream infection strains in the Library came from other hospitals and provinces. A total of 197 *E. anophelis* isolated from sputum (138 isolates), blood (25), abdominal fluid (7), cerebrospinal fluid (5), bronchoalveolar fluid (5), cardiac catheter (3), bile (2), pleural fluid (2), throat (2), burns (1), secretion (1), drainage (1), swab (1), body fluid (1), heart valve (1), abscess(1), and urine(1) were included in the work. For comparative genomic analysis, 318 *E. anophelis* strains were used in this study, including 197 newly sequenced and 121 publicly available strains (Supplementary Table 1), comprising the global collection isolates originating from more than 12 countries spanning four continents (Asia, Europe, Africa, and North America). At the time of writing this paper, only 121 whole-genome sequences of *E. anophelis* species, including raw data were available in the NCBI genome sequence repository of GenBank or SRA centre. Full details of the isolates (including download strains) are provided in Supplementary Table 1.

The *E. anophelis* we isolated were initially identified using a matrix-assisted laser desorption ionization time-of-flight mass spectrometry (MALDI–TOF MS) (Bruker Daltonics, USA), which may be misidentified. To reconfirm our 197 collected *E. anophelis* and reduce the impact on our collection, we constructed a heatmap based on the average nucleotide identity (ANI) values. Bacteria *E. anophelis* NUHP1 and R26, *E. meningoseptica* G4120 and KC1913, *E. miricola* EM798-26 and BM10 were as reference genomes.

### Genome sequencing, assembly and annotation

Bacteria were cultivated in Mueller–Hinton Broth (Oxoid, UK) without antibiotics in aerophilic conditions at 37°C on a shaker for 24–48 h. DNA extraction was performed using the Gentra Puregene Yeast/Bact. Kit (Qiagen, Germany) according to the manufacturer’s instructions. The harvested DNA was detected by the agarose gel electrophoresis and quantified by Qubit® 2.0 Fluorometer (Thermo Scientific).

A total amount of 1 µg of DNA per sample was used as input material for the DNA sample preparations. Following the manufacturer's recommendations, sequencing libraries were generated using NEBNext® Ultra™ DNA Library Prep Kit for Illumina (NEB, USA), and index codes were added to attribute sequences to each sample. PCR products were purified (AMPure XP system), and libraries were analysed for size distribution by Agilent 2100 Bioanalyzer and quantified using real-time PCR. The whole genome of *E. anophelis* was sequenced using Illumina NovaSeq PE150 at the Beijing Novogene Bioinformatics Technology Co., Ltd. To ensure the accuracy and reliability of the subsequent information analysis results, the Raw Data was filtered to obtain Clean Data. Assembled was performed with three different software: SOAP *denovo*, SPAdes and Abyss. Then, the three software assembly results were integrated with CISA software, and the assembly result with the least scaffolds was selected.

Genome component prediction included the prediction of the coding gene, repetitive sequences, non-coding RNA, genomics islands. We used the GeneMarkS [21] programme to retrieve the related coding gene. The interspersed repetitive sequences were predicted using the RepeatMasker [22] (http://www.repeatmasker.org/). The tandem Repeats were analyzed by the TRF (Tandem repeats finder) [23]. Transfer RNA (tRNA) genes were predicted by the tRNAscan-SE [24]. Ribosome RNA (rRNA) genes were analyzed by the rRNAmmer [25]. Small nuclear RNAs (snRNA) were predicted by BLAST against the Rfam database [26,27]. The IslandPath-DIOMB [28] programme was used to predict the genomics islands.

### Data analysis of the genomes

ANI values of all sequenced genomes were analyzed by FastANI [29]. To assess variation of the entire genome, including intergenic regions, for phylogenetic analysis of the global isolates, all de novo assemblies were aligned to the reference genome of *E. anophelis* CSID_3015183678, using MUMmer [30] (version 3.23). The Nucmer scripts are used for standard DNA sequence alignment to generate nucleotide alignments between two multi-FASTA input files with maximal exact matching. Extract 100 bp sequences on both sides of the single nucleotide polymorphism (SNP) site, and then use BLAST software to compare the extracted sequence with the assembly results to verify the single SNP site. Read mapping, SNP calling and preliminary filtering were completed using the RedDog phylogenomics pipeline (https://github.com/katholt/RedDog). Then, the concatenated alignment of these SNP alleles was used to generate a maximum likelihood phylogenetic tree for all the *E. anophelis* isolates using PhyML [31] with the HKY85 model. Different base substitution rates lead to the evolution of different species, the phylogenetic tree shows the evolutionary relationship and topological structure of different species. For the division principle of the cluster, it is divided according to the topological structure, the length and relationship between the branches. The tree was outgroup-rooted by including a pseudo sequence comprising all the alleles in the alignment. Support for the ML phylogeny was assessed via 1000 bootstrap pseudo-analyses of the alignment data. For the supermatrix of characters, the phylogenetic analysis was performed using IQ-TREE [32] with the evolutionary codon model being selected to minimize the BIC criterion. All trees were visualized and annotated using Evolview [33] and Python (https://github.com/katholt/plotTree/#python-code). The globally geographical distribution graph was performed by R software (version 3.5.3) with the rworldmap package. An interactive version of the global phylogeny labelled genotype, country origin, and isolation year was performed by Microreact [34].

*E. anophelis* ICEs structures (ICEEa) were predicted for all genome assemblies using ICEberg [35] database and confirmed by manual inspection using the following parameters. The annotation of each genome was searched for clusters of genes coding for an integrase, relaxase, coupling protein (T4CP), and transfer (Tra) proteins, including a VirB4 ATPase (TraG) in the conjugation module, which are the critical components of an ICE [36].

Temporal analysis of *E. anophelis* was performed with BEAST 2.5.2 [37]. Molecular tip-randomization analyses of the temporal signal in each analysis was initially assessed using an R package TipDatingBeast [38] based on 20 samples with reshuffled dates. Genomes of ICEEaI carriage *E. anophelis* were aligned to define a core and accessory genome using Roary [39]. The resulting alignment of 86, 818 core SNPs was used to deduce a RAxML phylogeny [40] using a general time reversible (GTR) evolutionary model. Timeline reconstruction and the most recent common ancestor (MRCA) determination of the ICEEaI carried *E. anophelis* cluster were performed using BEAST 2.5.2 [37], which model selection method was used nested sampling. Date estimates of all nodes were derived using BEAST on the SNP alignment, using tree clock models (relaxed log-normal, relaxed exponential, and strict clock models each combined with constant, exponential and coalescent Bayesian skyline population models). For model evaluation, the bModelTest module of BEAUti was used, and each combination was computed in three independent runs for ten million iterations, sampling every 5,000 steps. Nested sampling produces estimated of the marginal likelihood and standard deviation estimates (SD). The relaxed clock has a log marginal likelihood estimate of about −615533 and the SD is 1.06. Models that failed to converge based on visual inspection or had effective sampling size (ESS) values <200 for key parameters were discarded. Ultimately, the relaxed log-normal clock with GTR and skyline population models always converged and this model was finally used to estimate the clock rate and the time to MRCA. To calibrate the molecular clock, we used the sampling year of all sequences.

Population dynamics of the *E. anophelis* were estimated using a flexible coalescent Bayesian skyline incorporated in BEAST V2.5.2 [37], combined with the GTR model and a relaxed Log-normal clock. After the Beast analysis is complete, the tracer was used to analyze the log file and draw the Bayesian skyline plot.

## Results

### The ANI analysis of collected *E. anophelis*

The values of 203 representative *Elizabethkingia* species are presented in Figure S1. The ANI values ranged from 79% (between E. *meningoseptica*_G4120 and *E. anophelis* SKLX001947) to 100% (among a coup of newly sequenced *E. anophelis*). Clearly, the ANI values among all *E. anophelis* strains are >95.6% (Figure S1), suggesting that they are the same species according to the microbial taxonomy for species delineation (>95% cut-off for ANI) [41].

### Phylogenetic diversity and geographical distribution

**Figure 1.**
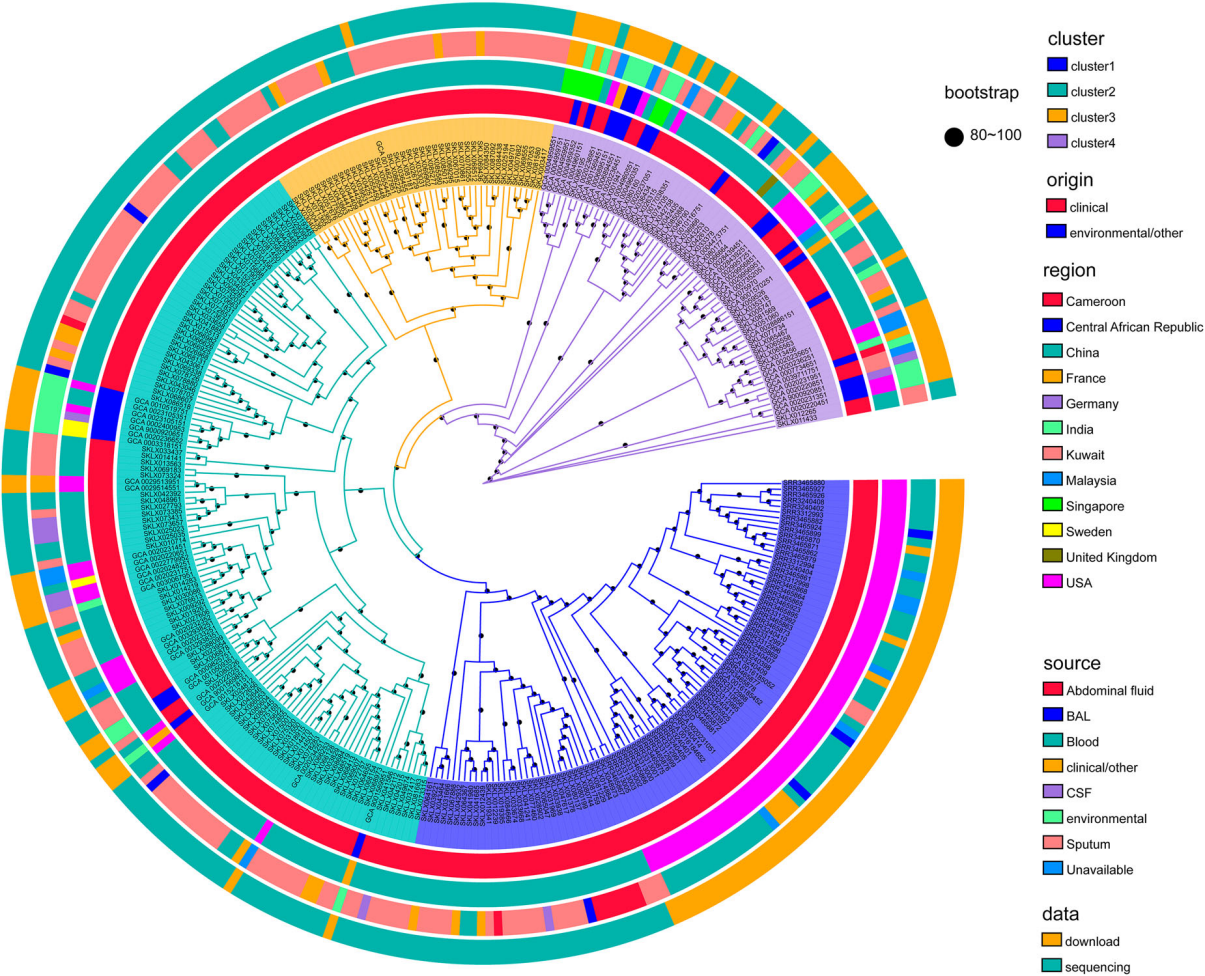
Population structure and the phylogenetic context of *E. anophelis*. Maximum likelihood tree based on single nucleotide polymorphisms of the 318 global isolates mapped against the reference strain *E. anophelis* CSID_3015183678. Primary clusters 1–4 are indicated in the inner coloured ring; branches defining these groups are coloured in the tree, and these groups are then divided into subclades. Coloured rings from the inside out indicate clusters, clinical or environmental isolates, country of origin, sample type and data sources, respectively. These rings are further divided into different colour combinations, representing a concrete definition. The black dots represent the bootstrap values. Origin: clinical or environmental isolates; Region: the country where the strain was isolated; Source: sample source of strain; BAL: bronchoalveolar fluid; CSF: cerebrospinal fluid.

To define the population diversity of the entire collection, a maximum likelihood phylogenetic tree was constructed based on SNPs in all global *E. anophelis* genomes (Figure 1). The primary observation was that our 197 contemporary Chinese sequences were distributed throughout this framework. The tree revealed four major branches, each containing several heterogeneous sub-clusters. Among these *E. anophelis* strains, there presented no closer clade of our sequenced isolate, even if cluster 3 merely contained stains from China. This structure tree demonstrated the evolutionary relationship of the strains had no special relationship with the sample source, origin and region (Figure 1). It might be an interesting observation that the result indicated the clinical *E. anophelis* bacteria only grouped in cluster 2 and cluster 4. It is worth our attention that cluster 1 not only contained all strains of the U.S. outbreak but has other Chinese clinical origin species. The circle map revealed those different origin strains had a high close phylogenetic kinship. Therefore, we speculate that Chinese-derived strains have the potential and risk of outbreaks and even global infections. Also, it indicated the potentially explosive *E. anophelis* strains were mainly from bloodstream and respiratory infections (Figure 1).

**Figure 2.**
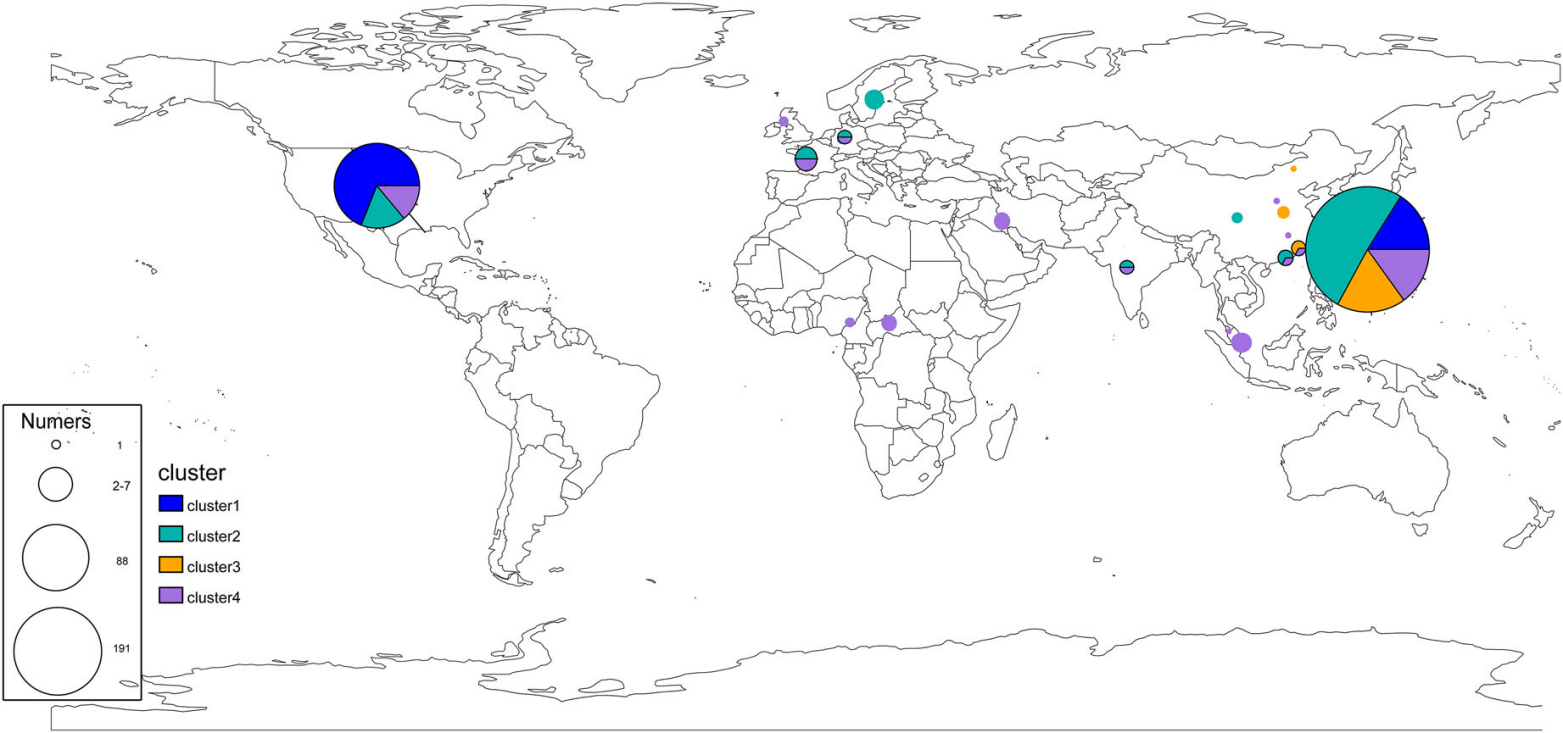
Geographical distribution and admixture of *E. anophelis* populations. Map of the world showed the subclade diversity existing of *E. anophelis* isolates in the global collection. Each circle indicates the population composition of a city/country, with a radius in proportion to the sample size. Pies are sized to indicate the number of isolates; slices are coloured by clusters. The clusters shared with the aforementioned result of clade membership of Figure 1. An interactive version of the global phylogeny, with organisms labelled by genotype, country of origin, and year of isolation, is available at https://microreact.org/project/r2egrptGKuEdK9mcZcCe5v.

Under the genotype and strain isolated region, we examined the globally geographical distribution of *E. anophelis* (Figure 2). For these analyses, isolates of the same clade and country were collapsed into a single representative pie (Figure 2). In addition, an interactive version of the global phylogeny, with organisms labelled by genotype, country of origin, and year of isolation, is available at Microreact (https://microreact.org/project/r2egrptGKuEdK9mcZcCe5v). The picture revealed the emerging pathogenic bacteria already distributed in various countries worldwide, especially in the USA and China. Primary clusters 1, 2, and 4 were broadly distributed across continents (blues, greens and purples, respectively, in Figure 2), likely reflecting the relatively ancient spread of *E. anophelis* across the globe. By providing expansive sampling across the continent, we observed a substantial degree of genetic diversity with multiple genotypes represented in 12 different economic levels countries (Figures 1 and 2). Conversely, all organisms belonging to genotype cluster 3 were found only in China sites (Figure 2). Interestingly, while the three common clusters (1, 2, 4) were present in most regions we analysed, cluster 2 predominated among American isolates (n = 61/88 unique isolates, 69%) (Figures 1 and 2). Furthermore, those unique isolates are exactly the threatening outbreak infection species (the USA experienced in 2015–2016). The population distribution also showed that the outbreak potential strain (Cluster 2) existed in America and China, revealing the possibility of a global outbreak spreading. Overall, these results demonstrate the robustness of localized *E. anophelis* outbreaks and the possibility of rapid global dissemination.

### Integrative and conjugative elements around global *E. anophelis*

Integrative and conjugative elements (ICEs), also called conjugative transposons, are a diverse group of mobile genetic elements found in both gram-positive and gram-negative bacteria, with a range of mechanisms to promote their core functions of integration, excision, transfer, and regulation, contributing to bacterial pathogenesis [42–44]. Recent research demonstrated that ICEs were ubiquitous in *E. anophelis* species, which were classified into three types: ICEEaI (Type I), ICEEaII (Type II) and ICEEaIII (Type III) [36].

**Figure 3.**
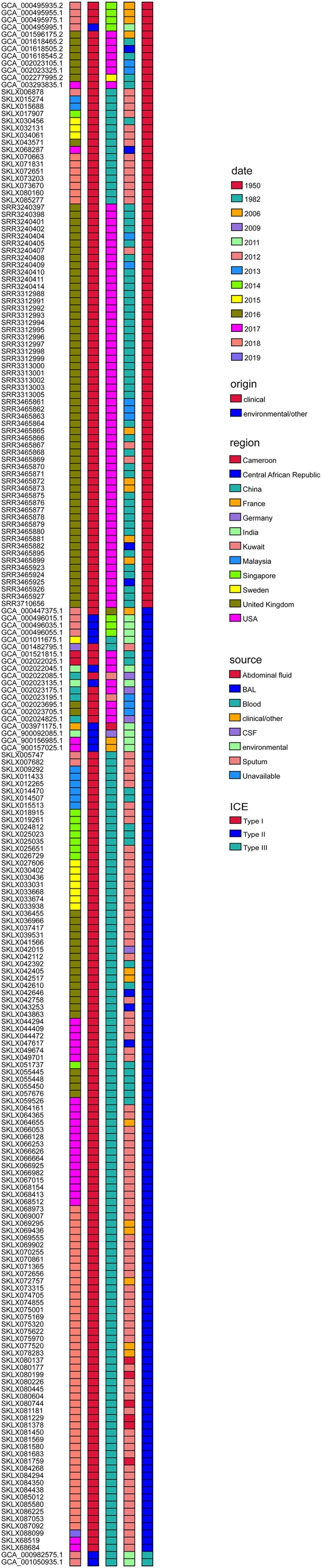
Distribution of Integrative and Conjugative Elements (ICEs) in *E. anophelis* strains isolated from around the world. The left-hand black columns represent the sample names of all isolates identified ICE. The coloured columns from left to right represent sample collected date (year), clinical or environmental isolates, country of origin, sample sources and ICE types, respectively. Type I: ICEEaI; Type II: ICEEaII; Type III: ICEEaIII. Different meaning of different colours in each concerned column has been detailedly interpreted on the rightmost.

In this research, all 318 genomes of *E. anophelis* strains worldwide were searched for ICEs (Figure 3). Based on the architecture of conjugation modules and associated signature genes, 217 ICEs were identified. The time-ICE heatmap revealed that 84 *E. anophelis* isolates had ICEEaI, and more than one hundred species contained ICEEaII while ICEEaIII merely recognized in two strains (Figure 3). The recognized ICE has no direct contact with the sample source (Figure 3). Nevertheless, the map revealed that the ICE type had a high relationship with the origin region of *E. anophelis*. Especially the ICEEaI mainly existed in the USA and China original strains, as well as several isolates in Singapore and Sweden (Figure 3). ICEEa1 consists of VirD4 ATPase (T4CP), relaxase, integrase, and several Tra proteins. Shockingly, this transposon element can insert into and disrupt the gene *MutY* (an adenine DNA glycosylase that is required to fix G-A mispairing), making the strain more liable to mutation and outbreak infection [13]. Indeed, the graph also showed that all USA outbreak *E. anophelis* strains contained Type I ICE (ICEEaI). Therefore, it implied the *E. anophelis* strains, outside the USA and especially in China, which contain ICEEaI might have the risks of localized outbreaks and rapid global dissemination. We should do a good job of monitoring and corresponding measures to prevent outbreaks of E. anophelis infection. It was an intriguing observation that among those Type I ICE possessed *E. anophelis* the original isolate came from China (in 2012), years earlier than the outbreak strain in the United States. Thus, there may be a possibility that the strain with the ability to break out of infection firstly originated in China and spread abroad.

### Bayesian maximum-clade-credibility time-scaled phylogenetic tree

**Figure 4.**
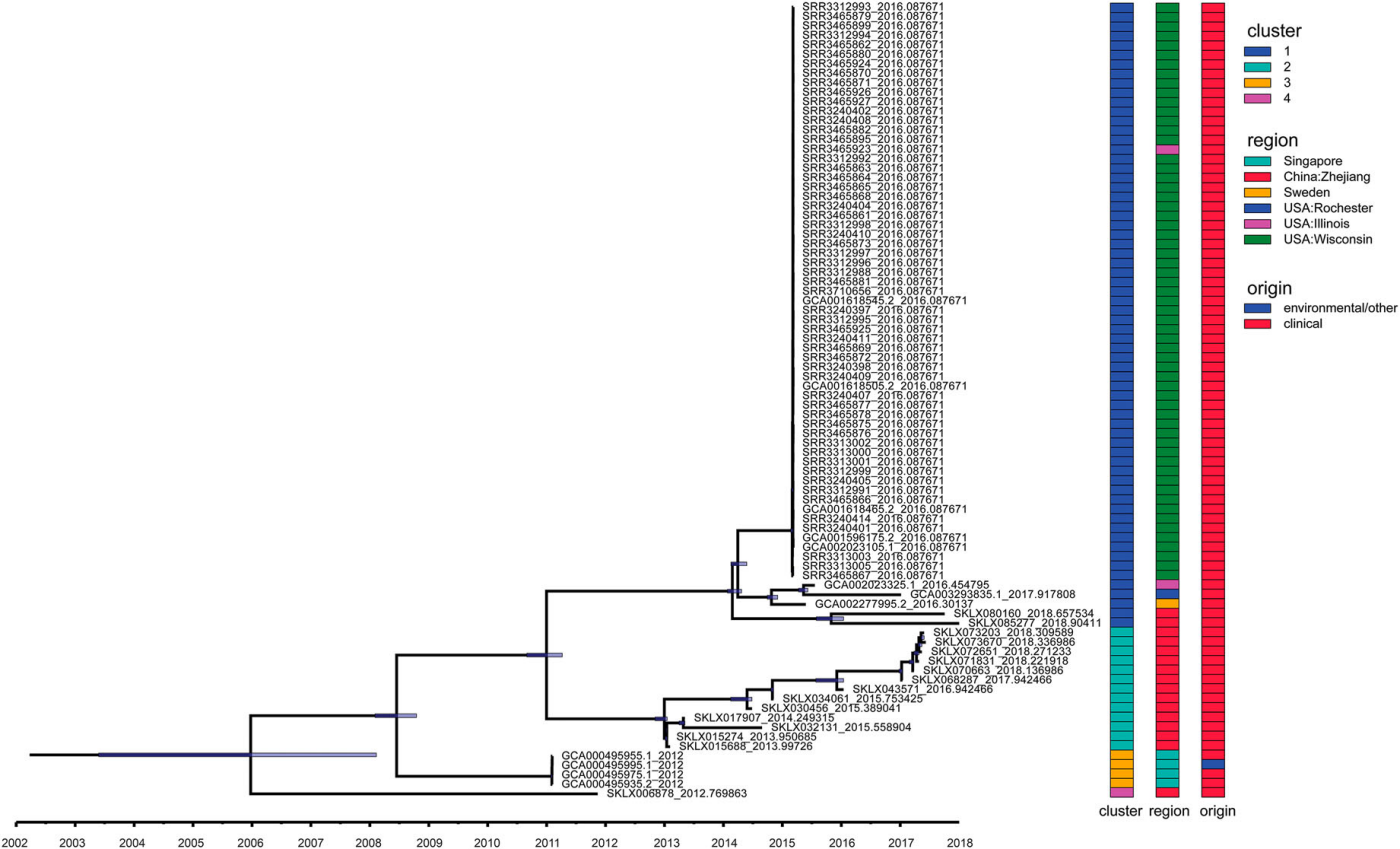
Temporal analysis on ICEEaI *E. anophelis* using BEAST (n = 84 isolates). Light blue horizontal bars centred on nodes indicate 95% highest probability density (HPD) values. (Right) Three different coloured bars denote cluster, origin and region respectively of these isolates. Clusters 1–4 are corresponding to Figure 1. Origin means clinical or environmental strains. Region indicates the country and place where the strain was isolated. Time bar (year) is shown at the bottom.

To investigate the structure and history of these 84 potentially explosive infectious *E. anophelis* pathogens (ICEEaI) emergence, we constructed temporally resolved phylogenies using Bayesian evolutionary analysis by sampling trees (BEAST 2.5.2) (Figure 4). Firstly, we tested whether there was a temporal signal, which indicating whether the root-to-tip distance was correlated with the real date of sampling of *E. anophelis* isolates. Bayesian analysis with tip-randomization test demonstrated a significant temporal signature (Figure S2), implying that the ICEEaI carriage *E. anophelis* strain continued diversifying in a measurable way over the course of the evolution. Based on the BEAST analysis, the MRCA for ICEEaI *E. anophelis* was dated twelve years ago (95% highest posterior density [HPD] interval, 2004–2008). The result suggests that China was the most likely recent source of this ICEEaI containing *E. anophelis* (Figure 4), which indicates a potential risk of an outbreak of infection. In the resulting recombination BEAST tree (Figure 4), the ICEEaI carriage *E. anophelis* isolates were divided into two well-supported lineages three times, which diverged around 2009, 2011, and 2014 respectively. Then all of the isolates belonged to each lineage were paraphyletic and formed several distinct, strongly supported subclades. The BEAST analysis also estimated that the American origin *E. anophelis* were formed into closed lineages (Figure 4). This most likely correlates with the emergence of the report of Perrin et al. [13].Using the Bayesian skyline model, we could estimate the effective *E. anophelis* population size in the past. The skyline plot revealed there were one major increases as well one decreases in the population size **(**Figure S3**)**. The increase began approximately seven years ago (2012), and the decreases occurred relatively close to three years ago (2016) but was lesser than the first rapid increase **(**Figure S3**)**. This observation is consistent with a previous report outbreak of infections in the United States between 2015 and 2016. If confidence intervals are taken into account, we assume that in this study we observed the last main increase in population size.

## Discussion

This large molecular epidemiology study showed how sequencing data could be used to gain insights into *E. anophelis* transmission networks across the globe. Within samples from various countries and different contemporary, we observed the emerging pathogen inherently has the possibility of global transmission and outbreak. To our knowledge, our study of *E. anophelis* is the largest and most representative in China to date and one of the largest internationally. We sampled isolates from a geographically dispersed subset of clinics and environments in various countries. Although we found some diﬀerences between the characteristics of the study isolates and those of all diagnoses in China, the absolute diﬀerences were small. Therefore, our study probably provides reliable insights into the molecular epidemiology or transmission of *E. anophelis* globally.

The species *E. anophelis* has recently emerged as a cause of life-threatening infections in humans, particularly in immunocompromised patients [5–10]. Several new species in the genus *Elizabethkingia* (including *E. bruuniana*, *E. ursingii*, *E. occulta*) have been proposed in the last decade [3]. Numerous studies have indicated that, rather than *E. meningoseptica*, *E. anophelis* is the most prevalent pathogen and has the highest mortality in this genus [4–10]. However, the identification of *Elizabethkingia* species remains a considerable challenge in clinical settings, and the cases of *E. anophelis* must be underestimated (especially in large population countries) due to the imperfect strain identification method (including biochemical-based phenotyping and MALDI–TOF MS systems). Indeed, in our research, nearly three hundred *E. anophelis* isolates were firstly identified by MALDI–TOF MS, but actually, only 197 were reconfirmed after genome sequencing and Nucleic acid alignment. In addition, our isolates number in is sustainable growth in recent years. Fortunately, recently, several studies have focused on the ameliorative identification means of *Elizabethkingia* species. It was reported MALDI–TOF MS systems with amended databases specific peaks could be used to differentiate *Elizabethkingia* species. However, these amended databases, either in the VITEK Mass Spectrometry or Bruker Biotyper systems, are merely available for research purposes but are not for clinical application in clinical microbiology laboratories [45]. Housekeeping gene sequencing has been increasingly used for microbial identification. Among the genotyping techniques of housekeeping gene sequencing, RNA polymerase β-subunit (*rpoB*) gene sequencing are able to correctly distinguish *Elizabethkingia* strains at the species level [3]. In addition, according to the documents, two polymerase chain reaction (PCR)-based methods, using uncommon primers or specific gene (*lepA*), have been recently developed for detecting and differentiating *Elizabethkingia* species [46,47].

In 2015–2016, the first shocking outbreak of infection of *E. anophelis* occurred in the USA. Unexpected, the case fatality rate was reported high at 31% (20/65) [9,18]. In addition, several small outbreaks of the bacterium in Singapore, Hong Kong, Taiwan, the USA, and South Korea had also been documented [8,10,12,15]. Intriguingly, the chief culprit causing *E. anophelis* outbreak in the USA was definitely attributed to the ICEs [13]. Here, what should pay attention to is that if there is or not the similar ICEs in another country's original *E. anophelis* strain? Unfortunately, after *in silico* identification of ICEs, we found the critical pathogenic type I ICE (ICEEaI) also existed in a part of our *E. anophelis*. It sounded the alarm for us that the *E. anophelis* might also have the ability to cause the outbreak of infections in China and rapid global dissemination. Expected, if the *E. anophelis* casing rapid global transmission and outbreaks, the damage will be more serious and invaluable. Therefore, it is necessary to monitor the epidemiology and transmission of the emerging bacterium to control and prevent the global outbreak of infections of the *E. anophelis*.

It is documented that *E. anophelis* has the character of multi-drug resistance, which show different degree of antibiotic resistance such as β-lactams, aminoglycosides, quinolones, tetracyclines, chloramphenicol, even carbapenems and vancomycin [4,7,9,10,16,17,48]. However, there have been few studies on *E. anophelis* resistance, and the multiple resistance mechanisms are still unclear. In recent years, biofilm has increasingly become a hot topic. Generally, biofilms are defined as a community of cells or bacteria encased within an exopolymeric matrix and attached to a surface. They are recognized as being more resistant to antimicrobial therapy and host defences [49,50]. Studies have shown that biofilms can make the bacteria enter a slow growth lag period, protecting them and gaining antibiotic resistance [51–54]. Intriguingly, we found all the researched *E. anophelis* contained multiple biofilm-forming genes such as *flmH*, *ugd*, *capL*, *rmlC*, *capE*, *fleQ*, *cpsO*, *cap8E*, *fnlA*, *VipB/tviC*, *uppS*, *motD*, *fleR*, *kpsF*, *wbjD*/*WecB*, *rmlD*, *rmlB*, *capL*, and *cap5H* (Figure S4). Therefore, we propose the following hypothesis: The related genes carried by *E. anophelis* bacterium promote the formation of a biofilm, then enter a slow growth lag phase, and acquire antibiotic multiple resistance characteristics. It may contribute to clarifying the antibiotic multiple resistance mechanisms of *E. anophelis*. Therefore, more and further experiments are deserved to perform.

A limitation of this study exists. We did not establish the true extent of the global distribution of *E. anophelis* because of obvious sampling bias (i.e. the available historical isolates studied here were predominantly from China and USA). This limitation also hinders our ability to account for secular trends. Nevertheless, an important reason is that many *E. anophelis* strains are not sequenced, and the available genome is limited. Apparently, available genomic data is far less than the number of reported cases. This indicates that people should pay enough attention to this emerging pathogenic bacterium in the future.

In conclusion, genomic, spatial, phylogenetic, and epidemiological data helped us to better understand the complex dynamics of *E. anophelis* transmission. Our study sheds light on the potential possibility of *E. anophelis* pathogens causing the large-scale outbreak and rapid global dissemination. Continued genomic surveillance of the dynamics of *E. anophelis* populations with increased geographical representation and length sampling time will generate further knowledge for optimizing future prevent outbreak infections and rapid global dissemination.

## Supplementary Material

Supplemental Material

## Data Availability

Accession numbers for all genome data included in this work are summarized in Supplementary Table 1. Every genome sequence assembled for the 197 newly sequenced *E. anophelis* isolates has been deposited in the NCBI, associated with project ID PRJNA643387. The software for the Microreact interactive tree viewer is available at: https://microreact.org/project/r2egrptGKuEdK9mcZcCe5v. Python script to visualize and annotate trees is available at https://github.com/katholt/plotTree/#python-code.
