# Supplementary figures and images for: Population genomics of emerging Elizabethkingia anophelis pathogens reveals potential outbreak and rapid global dissemination
Source: Emerg Microbes Infect. 2022 Nov 4;11(1):2590–9. doi: 10.1080/22221751.2022.2132880 (PMC9639502; doi:10.1080/22221751.2022.2132880)

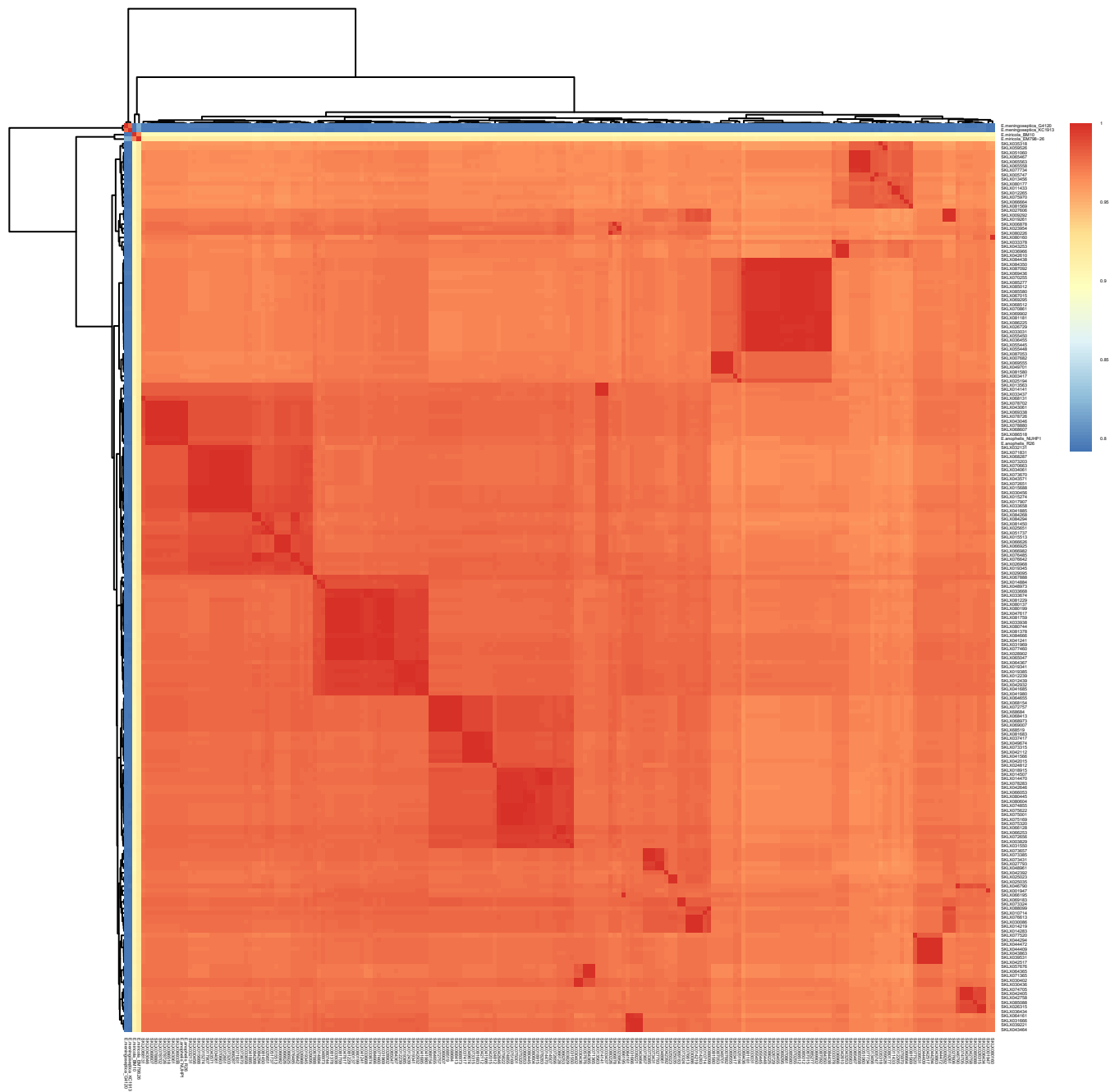

Supplement: Supplemental Material [file TEMI_A_2132880_SM3689.zip › Figure S1.pdf]

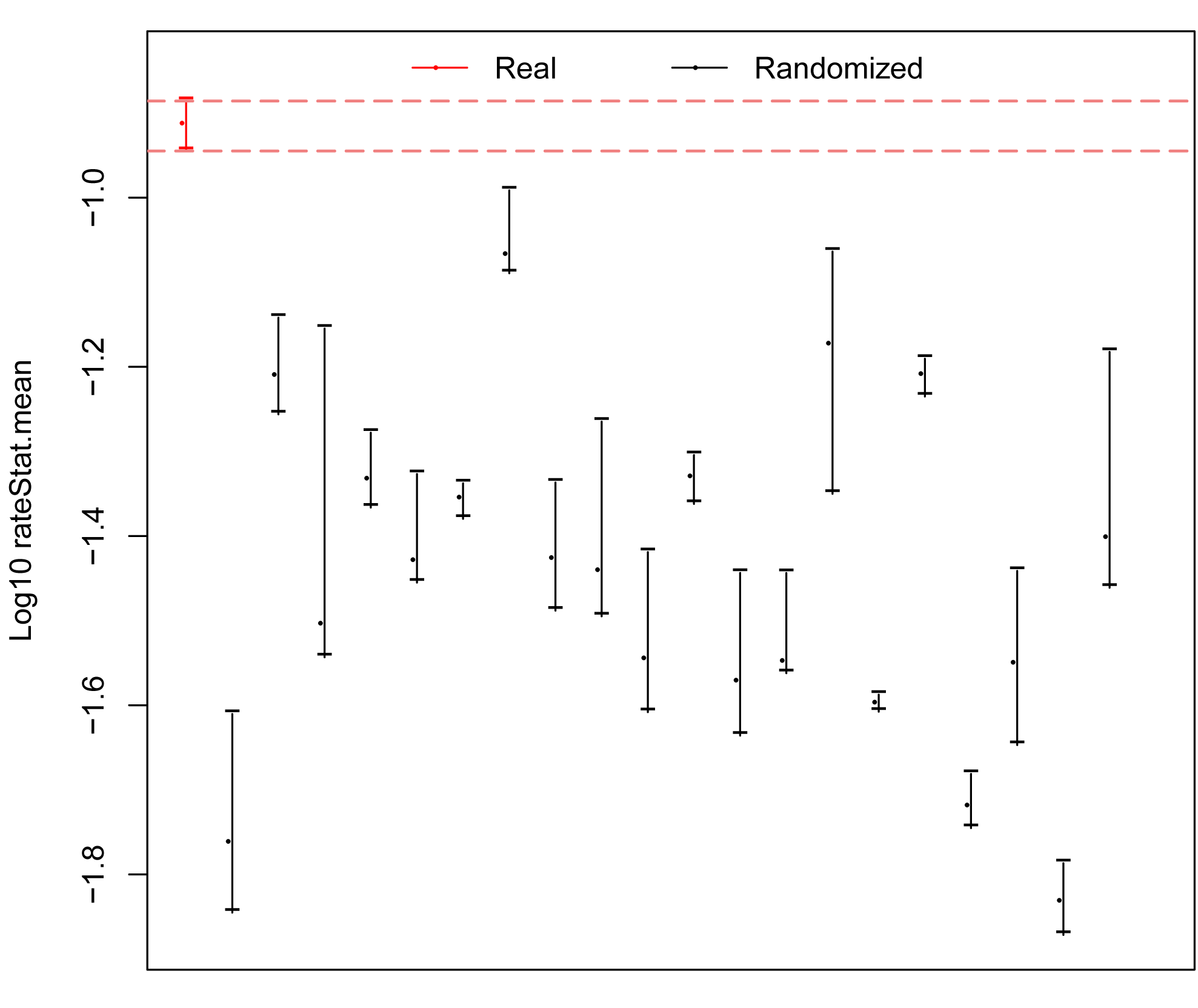

Supplement: Supplemental Material [file TEMI_A_2132880_SM3689.zip › Figure S2.tif]

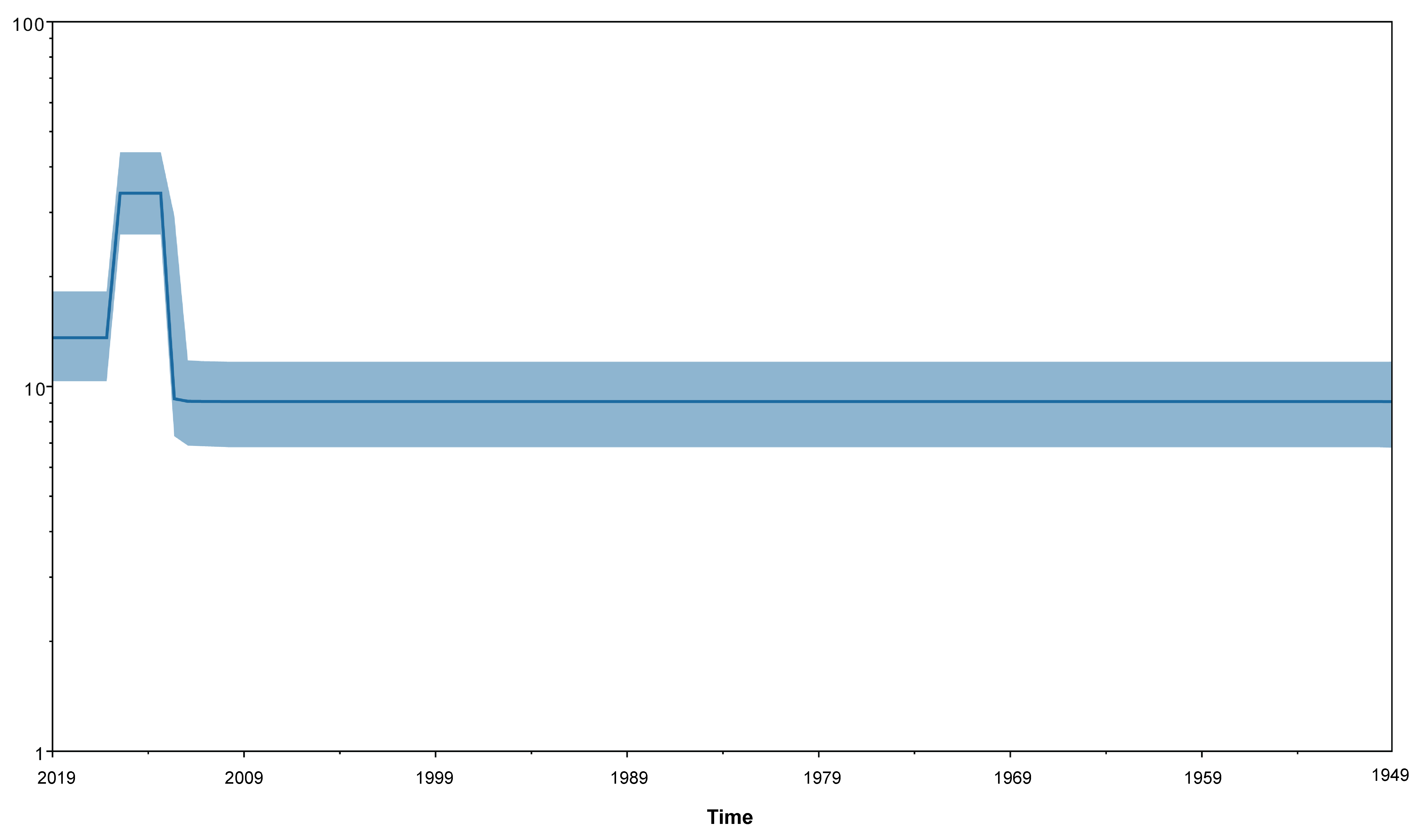

Supplement: Supplemental Material [file TEMI_A_2132880_SM3689.zip › Figure S3.tif]

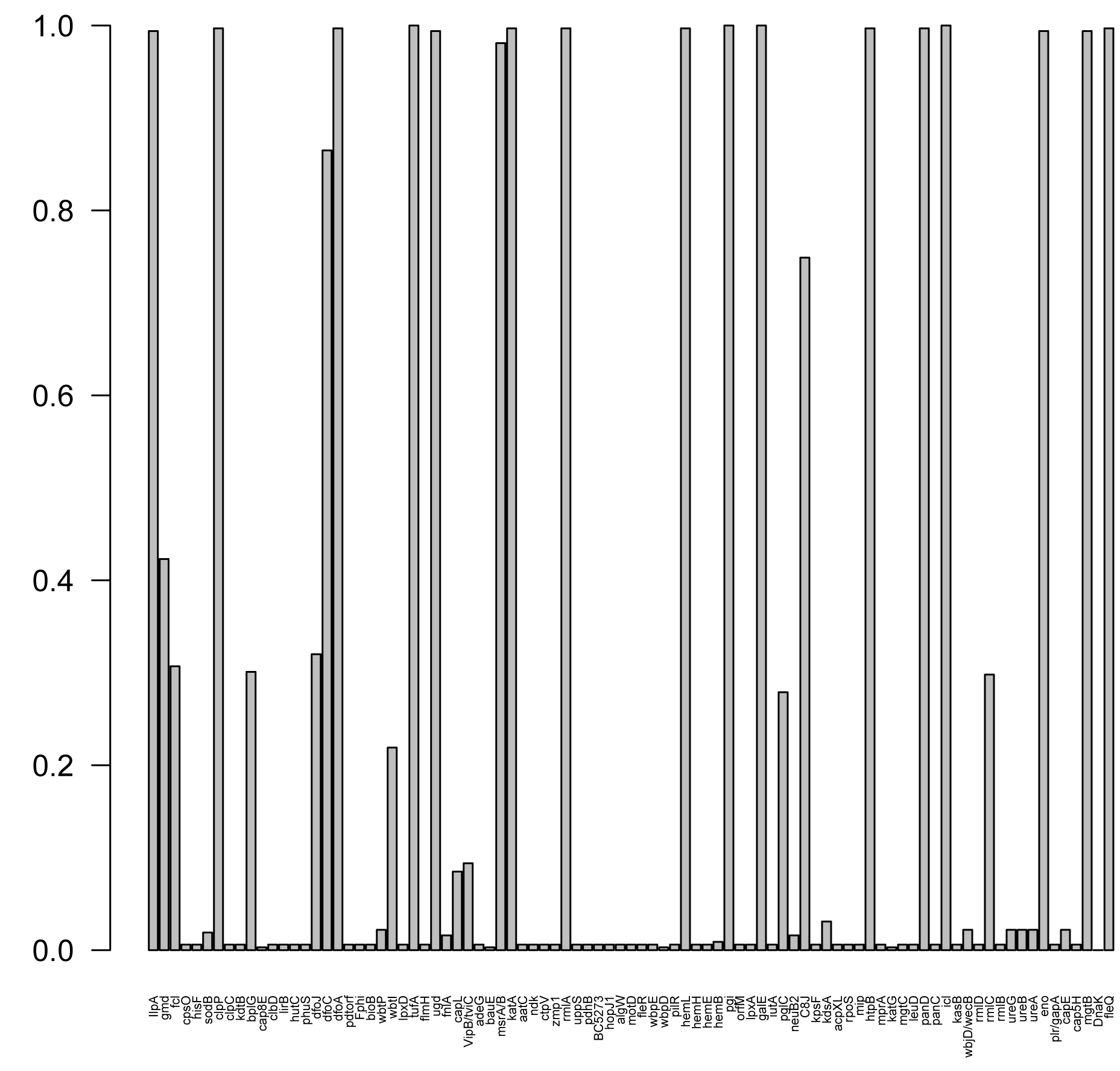

Supplement: Supplemental Material [file TEMI_A_2132880_SM3689.zip › Figure S4.tif]
